# Supplementary material for: The Complex Interplay between the Gut Microbiome and Osteoarthritis: A Systematic Review on Potential Correlations and Therapeutic Approaches
Source: Int J Mol Sci. 2023 Dec 21;25(1):143. doi: 10.3390/ijms25010143 (PMC10778637; doi:10.3390/ijms25010143)
Supplement: Supplementary file 1 [file ijms-25-00143-s001.zip › ijms-2733485-supplementary.pdf]

**Table S1.** PRISMA 2020 Checklist

| Section and Topic | Item # | Checklist item | Location where item is reported |
|---|---|---|---|
| **TITLE** | | | |
| Title | 1 | Identify the report as a systematic review. | Lines 2-4 |
| **ABSTRACT** | | | |
| Abstract | 2 | See the PRISMA 2020 for Abstracts checklist. | Lines 11-32 |
| **INTRODUCTION** | | | |
| Rationale | 3 | Describe the rationale for the review in the context of existing knowledge. | Lines 36-114 |
| Objectives | 4 | Provide an explicit statement of the objective(s) or question(s) the review addresses. | Lines 114-117 |
| **METHODS** | | | |
| Eligibility criteria | 5 | Specify the inclusion and exclusion criteria for the review and how studies were grouped for the syntheses. | Lines 128-142 |
| Information sources | 6 | Specify all databases, registers, websites, organisations, reference lists and other sources searched or consulted to identify studies. Specify the date when each source was last searched or consulted. | Lines 144-156 |
| Search strategy | 7 | Present the full search strategies for all databases, registers and websites, including any filters and limits used. | Lines 128-151 |
| Selection process | 8 | Specify the methods used to decide whether a study met the inclusion criteria of the review, including how many reviewers screened each record and each report retrieved, whether they worked independently, and if applicable, details of automation tools used in the process. | Lines 159-165 |
| Data collection process | 9 | Specify the methods used to collect data from reports, including how many reviewers collected data from each report, whether they worked independently, any processes for obtaining or confirming data from study investigators, and if applicable, details of automation tools used in the process. | Lines 166-180 |
| Data items | 10a | List and define all outcomes for which data were sought. Specify whether all results that were compatible with each outcome domain in each study were sought (e.g. for all measures, time points, analyses), and if not, the methods used to decide which results to collect. | Lines 166-180 |
| | 10b | List and define all other variables for which data were sought (e.g. participant and intervention characteristics, funding sources). Describe any assumptions made about any missing or unclear information. | Lines 166-180 |
| Study risk of bias assessment | 11 | Specify the methods used to assess risk of bias in the included studies, including details of the tool(s) used, how many reviewers assessed each study and whether they worked independently, and if applicable, details of automation tools used in the process. | Lines 181-188 |
| Effect measures | 12 | Specify for each outcome the effect measure(s) (e.g. risk ratio, mean difference) used in the synthesis or presentation of results. | Lines 167-180 |
| Synthesis methods | 13a | Describe the processes used to decide which studies were eligible for each synthesis (e.g. tabulating the study intervention characteristics and comparing against the planned groups for each synthesis (item #5)). | Lines 128-142 |
| | 13b | Describe any methods required to prepare the data for presentation or synthesis, such as handling of missing summary statistics, or data conversions. | Lines 167-180 |

| Section and Topic | Item # | Checklist item | Location where item is reported |
|---|---|---|---|
| | 13c | Describe any methods used to tabulate or visually display results of individual studies and syntheses. | Lines 167-180 |
| | 13d | Describe any methods used to synthesize results and provide a rationale for the choice(s). If meta-analysis was performed, describe the model(s), method(s) to identify the presence and extent of statistical heterogeneity, and software package(s) used. | Lines 167-180 |
| | 13e | Describe any methods used to explore possible causes of heterogeneity among study results (e.g. subgroup analysis, meta-regression). | Not applicable |
| | 13f | Describe any sensitivity analyses conducted to assess robustness of the synthesized results. | Not applicable |
| Reporting bias assessment | 14 | Describe any methods used to assess risk of bias due to missing results in a synthesis (arising from reporting biases). | Lines 181-188 |
| Certainty assessment | 15 | Describe any methods used to assess certainty (or confidence) in the body of evidence for an outcome. | Not applicable |
| **RESULTS** | | | |
| Study selection | 16a | Describe the results of the search and selection process, from the number of records identified in the search to the number of studies included in the review, ideally using a flow diagram. | Lines 190-197 |
| | 16b | Cite studies that might appear to meet the inclusion criteria, but which were excluded, and explain why they were excluded. | Lines 191-195 |
| Study characteristics | 17 | Cite each included study and present its characteristics. | Lines 202-291, 303-336 |
| Risk of bias in studies | 18 | Present assessments of risk of bias for each included study. | Lines 342-358 |
| Results of individual studies | 19 | For all outcomes, present, for each study: (a) summary statistics for each group (where appropriate) and (b) an effect estimate and its precision (e.g. confidence/credible interval), ideally using structured tables or plots. | Not applicable |
| Results of syntheses | 20a | For each synthesis, briefly summarise the characteristics and risk of bias among contributing studies. | Lines 293-302, 337-341 |
| | 20b | Present results of all statistical syntheses conducted. If meta-analysis was done, present for each the summary estimate and its precision (e.g. confidence/credible interval) and measures of statistical heterogeneity. If comparing groups, describe the direction of the effect. | Not applicable |
| | 20c | Present results of all investigations of possible causes of heterogeneity among study results. | Lines 202-291, 303-336 |
| | 20d | Present results of all sensitivity analyses conducted to assess the robustness of the synthesized results. | Not applicable |
| Reporting biases | 21 | Present assessments of risk of bias due to missing results (arising from reporting biases) for each synthesis assessed. | Lines 342-368 |

| Section and Topic | Item # | Checklist item | Location where item is reported |
|---|---|---|---|
| Certainty of evidence | 22 | Present assessments of certainty (or confidence) in the body of evidence for each outcome assessed. | Not applicable |
| **DISCUSSION** | | | |
| Discussion | 23a | Provide a general interpretation of the results in the context of other evidence. | Lines 369-422 |
| | 23b | Discuss any limitations of the evidence included in the review. | Lines 423-441 |
| | 23c | Discuss any limitations of the review processes used. | Lines 423-441 |
| | 23d | Discuss implications of the results for practice, policy, and future research. | Lines 443-460 |
| **OTHER INFORMATION** | | | |
| Registration and protocol | 24a | Provide registration information for the review, including register name and registration number, or state that the review was not registered. | In progress |
| | 24b | Indicate where the review protocol can be accessed, or state that a protocol was not prepared. | Protocol was not prepared |
| | 24c | Describe and explain any amendments to information provided at registration or in the protocol. | None |
| Support | 25 | Describe sources of financial or non-financial support for the review, and the role of the funders or sponsors in the review. | Line 467 |
| Competing interests | 26 | Declare any competing interests of review authors. | Line 472 |
| Availability of data, code and other materials | 27 | Report which of the following are publicly available and where they can be found: template data collection forms; data extracted from included studies; data used for all analyses; analytic code; any other materials used in the review. | Line 470 |

*From:* Page MJ, McKenzie JE, Bossuyt PM, Boutron I, Hoffmann TC, Mulrow CD, et al. The PRISMA 2020 statement: an updated guideline for reporting systematic reviews. BMJ 2021;372:n71. doi: 10.1136/bmj.n71

**Table S2:** SYRCLE's tool for assessing risk of bias in the in vivo studies.

| SYRCLE | | | | | | | | | | |
|---|---|---|---|---|---|---|---|---|---|---|
| **Study** | **Selection bias** | | | **Performance bias** | | **Detection bias** | | **Attrition bias** | **Reporting bias** | **Other** |
| | **Sequence generation** | **Baseline characteristics** | **Allocation concealment** | **Random housing** | **Blinding** | **Random out come assessment** | **Blinding** | **Incomplete outcome data** | **Selective outcome reporting** | **Other sources of bias** |
| Lan et al. 2021 | | | | | | | | | | |
| Guan et al. 2020 | | | | | | | | | | |
| O-Sullivan et al. 2022 | | | | | | | | | | |
| Zhang et al. 2022 | | | | | | | | | | |
| Collins et al. 2015 | | | | | | | | | | |
| Yan et al. 2021 | | | | | | | | | | |
| Li et al. 2021 | | | | | | | | | | |
| Xu et al. 2022 | | | | | | | | | | |
| Cho et al. 2022 | | | | | | | | | | |
| Guss et al. 2019 | | | | | | | | | | |

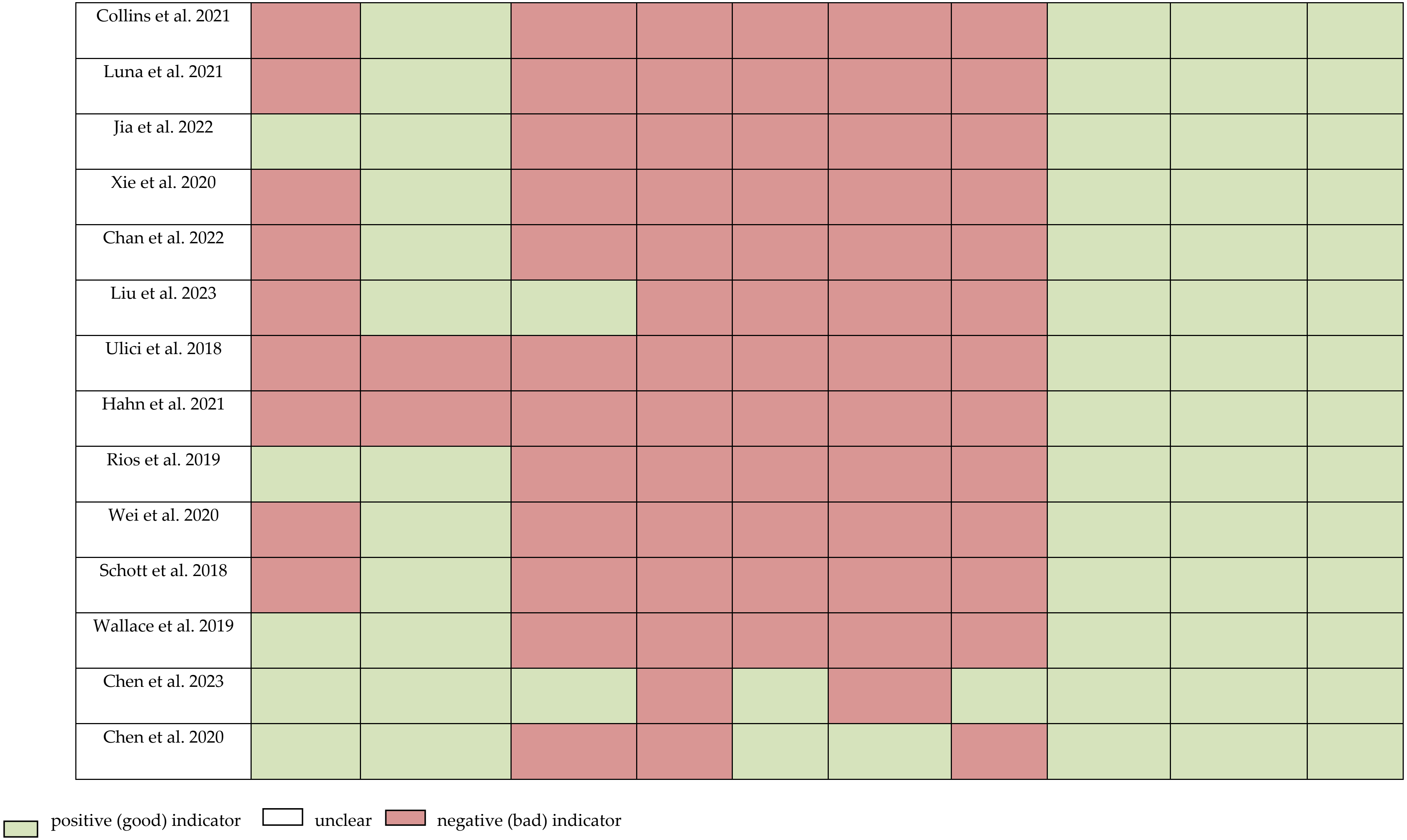
Collins et al. 2021
Luna et al. 2021
Jia et al. 2022
Xie et al. 2020
Chan et al. 2022
Liu et al. 2023
Ulici et al. 2018
Hahn et al. 2021
Rios et al. 2019
Wei et al. 2020
Schott et al. 2018
Wallace et al. 2019
Chen et al. 2023
Chen et al. 2020
positive (good) indicator
unclear
negative (bad) indicator

**Table S3:** ROBINS-I tool for assessing risk of bias in non-randomized clinical studies.

| ROBINS-I | | | | | | | | |
|---|---|---|---|---|---|---|---|---|
| Study | | Pre-intervention | | At intervention | Post-intervention | | | |
| First author | Year | Bias due to confounding | Bias in selection of participants into the study | Bias in classification of interventions | Bias due to deviations from intended interventions | Bias due to missing data | Bias in measurement of outcomes | Bias in selection of the reported result |
| Yu et al. | 2021 | low | moderate | low | low | low | low | low |
| Wang et al. | 2021 | low | low | low | low | low | low | moderate |
| Boer et al. | 2019 | low | low | low | low | low | low | moderate |
| Chen et al. | 2023 | low | low | low | low | moderate | low | low |
| Ramasamy et al. | 2021 | low | low | low | low | low | low | low |
| Guan et al. | 2023 | low | low | low | low | low | low | low |
| Rushing et al. | 2022 | low | low | low | low | low | low | moderate |
| Pedersini et al. | 2021 | low | low | low | low | low | moderate | low |
| Chen et al. | 2021 | low | low | low | low | low | low | low |
| Wang et al. | 2021 | low | low | low | low | low | low | low |
| Arbeeva et al. | 2022 | low | low | low | low | low | low | moderate |
| Lee et al. | 2021 | low | low | low | low | low | low | moderate |
| Taye et al. | 2020 | low | low | low | low | moderate | low | low |
| Wang et al. | 2023 | low | low | low | low | low | low | low |

low moderate serious
